# Epigenetic Regulation and Nonepigenetic Mechanisms of Ferroptosis Drive Emerging Nanotherapeutics in Tumor

**DOI:** 10.1155/2021/8854790

**Published:** 2021-01-29

**Authors:** Yue Cheng, Yao Xie, Yan Chen, Xiaojing Liu

**Affiliations:** ^1^Department of Cardiology, West China Hospital, Sichuan University, Chengdu 610041, China; ^2^State Key Laboratory of Biotherapy and Cancer Center, West China Hospital, Sichuan University, Chengdu 610041, China; ^3^Laboratory of Cardiovascular Diseases, Regenerative Medicine Research Center, West China Hospital, Sichuan University, Chengdu 610041, China

## Abstract

Currently, traditional cancer therapy still falls far short of expectations. However, a variety of invasive cancers that are resistant to chemotherapy (such as platinum drugs, one of the most applied antineoplastics in clinic) and targeted agents are susceptible to ferroptosis. Ferroptosis is a form of cell death that is driven by cell metabolism and iron-dependent lipid peroxidation. Ferroptosis inducers can eliminate the drug resistance of tumor cells in the mesenchymal state, effectively inhibit the drug resistance of acquired tumor cells, and optimize cancer efficacy. Research based on the epigenetic mechanism of ferroptosis is still in the stage of screening and verifying the regulatory effect, and there is no complete regulatory mechanism network. In this review, we expound on the epigenetic regulation and nonepigenetic mechanisms of ferroptosis and review the epigenetic-based mechanisms of tumor therapy potential and emerging nonepigenetic-based therapies (nanotherapeutics).

## 1. Introduction

The occurrence of cancer is interrelated with human genes. Mainstream tumor therapy includes surgery, gene therapy (targeted therapy), and immune-related therapy. Surgery is mainly effective for carcinoma in situ and is prone to recurrence after surgery. Targeted therapy and immunotherapy are effective for some patients, but most patients fail due to the emergence of drug resistance [1]. In the global medical field, much research has focused on the breakthrough in overcoming drug resistance during targeted tumor therapy. In general, all therapies are designed to inhibit tumor cell proliferation and/or promote tumor cell death. The latter is based on inducing regulatory cell death (RCD), which includes apoptosis, pyroptosis, reticulation, invagination, and autophagy [2]. Ferroptosis is an important form of RCD, caused by the accumulation of lipid free radicals dependent on iron ions, and is lucubrated more extensively in the mechanism of cancer treatment [3]. Ferroptosis is involved in the occurrence and evolution of a variety of diseases, such as tumor [4], ischemia reperfusion injury (IRI) [5], renal failure [6], neurological diseases [7], and hematological diseases [8]. Especially in cancer research, ferroptosis can induce the death of various tumor cells, including in liver cancer, pancreatic cancer, and breast cancer, but its mechanism remains to be further explored [9–12]. While new research on epigenetics is increasing, the mechanism and application of ferroptosis in the treatment of tumors have become clearer. Without changing the DNA sequence, epigenetics can cause heritable gene expression or cell phenotype changes through certain mechanisms.

In addition, the application of nanotechnology in the biomedical fields has been expanded in both depth and breadth with the rapid development of material science. Nanotechnology has the advantages of strong targeting, low system toxicity, controllable drug release performance, and synergistic effects of novel emerging compound therapies and development of targeting research. It can improve the solubility of drugs, prolong plasma half-life, and promote cell internalization and enhancement accumulation at the tumor site, thereby providing some possibilities to eradicate drug-resistant cancer cells [13]. The combination of nanotechnology and ferroptosis has broad possibilities for the treatment of tumor, especially for patients with weak response or drug resistance to cancer treatment. Therefore, we expound on the mechanism and outline the epigenetic effects of ferroptosis and further optimize the treatment of drug-resistant cancer by combining emerging medical technologies with traditional therapies.

## 2. Mechanism of Ferroptosis

**Figure 1:**
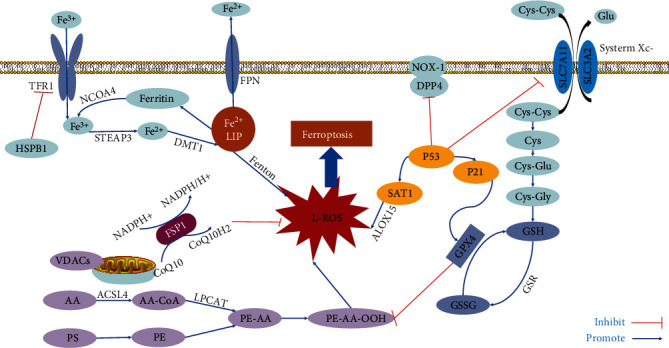
This figure summarizes the regulatory core of ferroptosis: (1) amino acid metabolism, including the X^C−^/GSH/GPX4 pathway; (2) mitochondrial mechanism, the FSP1-CoQ10-NAD(P) H pathway; (3) iron metabolism, including HSPB1-TFR1, the NCOA4 pathway, and the DMT1 pathway; (4) lipid metabolism, ACSL4, and LPCAT3; (5) glucose metabolism, DPP4-NOX1 complex; (6) P53-mediated metabolism, including the P53-SAT1-ALOX15 pathway and the P53-SLC7A11-GSH pathway. TFR1: transferrin receptor 1; NCOA4: nuclear receptor coactivator 4; STEAP3: six transmembrane epithelial antigen of the prostate 3; DMT1: divalent metal transporter 1; HSPB1: heat shock protein beta-1; FPN: ferroportin; LIP: labile iron pool; ACSL4: acyl-CoA synthetase long-chain family member 4; AA: arachidonoyl; NADPH: nicotinamide adenine dinucleotide phosphate; CoQ10: coenzyme Q10; VDAC: voltage-dependent anion channel; FSP1: ferroptosis suppressor protein 1; PS: phosphatidylserine; PE: phosphatidylethanolamine; L-ROS: lipid reactive oxygen species; Cys: cysteine; Glu: glutamate; Gly: glycine; GSH: glutathione; GPX4: glutathione peroxidase 4; GSSG: oxidized glutathione; ACSL4: acyl-CoA synthetase long-chain family member 4; ALOX: arachidonate lipoxygenase; AA: arachidonoyl; AdA: adrenoyl; ABCB6: ATP-binding cassette subfamily B member 6; ATG5: autophagy-related 5; ATG7: autophagy-related 7; CoQ10: coenzyme Q10; Cys: cysteine; system Xc-: cysteine/glutamate transporter receptor; GSR: glutathione-disulfide reductase; SAT1: spermidine/spermine N1-acetyltransferase 1.

The phenomenon of ferroptosis was first discovered by Sonam [14] in 2003 and first named by Dixon [15] in 2012. The content of ferritic ions is significantly increased, which generates a massive consumption of glutathione (GSH) and the inactivation of glutathione peroxidase 4 (GPX4). The oxidation of lipoxygenase results in more polyunsaturated fatty acids (PUFAs), cholesterol, and phospholipids, eventually causing lipid peroxidation and the production of excessive reactive oxygen species (ROS). Lipid peroxides form aldol compounds such as malondialdehyde, which induces cell membranes to rupture and die [16]. Ferroptotic cells present no ruptured plasma membrane, rounded cells, shrunken cristae, normal nuclei, or uncondensed chromatin [17]. Various molecules and signals involved in the accumulation of iron and subsequent lipid peroxidation are the key to regulating ferroptosis. Of nonepigenetic mechanisms and epigenetic regulation, the former is based on a combination of ferroptosis metabolic networks (Figure 1), while the latter is based on genome-level epigenetic changes. Both can change the sensitivity to ferroptosis [18].

### 2.1. Nonepigenetic Mechanisms

Amino acid metabolism, mitochondrial mechanisms, iron metabolism, lipid metabolism, glucose metabolism, and P53 mechanism are nonepigenetic mechanisms of ferroptosis (Figure 1).

#### 2.1.1. Amino Acid Metabolism

System X^C-^ is a reverse transport protein located on the cell membrane. It is a heterodimer composed of the light chain subunit XCT encoded by SLC7A11 as the substrate-specific subunit and the heavy chain encoded by SLC3A2. It can transport glutamate out of the cell in a certain proportion, transport cysteine into the cell, and use cysteine as a raw material to synthesize glutathione GSH [19]. The sulfhydryl structure in GSH can be oxidized and dehydrogenated, making GSH an important antioxidant and free radical scavenger in the body. Reducing the synthesis of GSH can reduce the ferroptotic effect. Inhibiting the activity of the X^C-^ system and reducing the raw material for GSH synthesis by inhibiting the transport of cystine will decrease intracellular GSH, which leads to a decrease in the activity of GPX4. GPX4 is a selenoprotein that inserts selenocysteine through the transporter selenocysteine transfer RNA (Sec-tRNA), effectively reduces peroxidized phospholipids [20–22], and inhibits the activation of arachidonic acid (AA) metabolic enzyme 6 [23]. GPX4 has a wider substrate preference and can react directly with LOOH on the membrane to change the integrity and biophysical properties of the membrane, transform the position or function of membrane related proteins, and degrade into highly active products, which may contribute to increasing membrane permeability and direct cytotoxicity [24]. The accumulation of LOOHs on the mitochondrial membrane can increase permeability, which explains mitochondrial swelling and rupture of the outer membrane [18]. GPX4 is the only enzyme described so far that can directly reduce complex phospholipid hydroperoxides. It can convert reduced GSH to oxidized GSH, reduce lipid peroxides to lipid alcohols, and convert hydrogen peroxide to water, which resists lipid peroxidation that depends on iron and O_2_. Antioxidants and iron chelating agents can inhibit ferroptosis. They regulate the production and degradation of L-ROS, leading to the loss of balance in this process and ultimately ferroptosis [15, 17]. To ensure the integrity of the membrane and minimize the damage caused by ROS, reduced GSH is used as a cofactor to convert lipid hydroperoxide (R-OOH) into lipid alcohol (R-OH) [18]. Redox GSH is an ideal substrate for GPX4, an indispensable substance for the prevention of death. Glutathione usually changes between the reduced state (GSH) and the oxidized state (GSSG). Oxidized GSSG is reduced through the process of converting NADPH to NADP+. Hence, the GSH/GSSG ratio reflects the degree of cell oxidation [25]. Some reports associate GPX4 activity with sensitivity to apoptosis, necrosis, and ferroptosis.

#### 2.1.2. Mitochondrial Mechanism

Voltage-dependent anion channels (VDACs) are ion channels located outside the mitochondrial membrane, and the main structure is a porous protein spanning the *β*-barrel structure of the mitochondrial membrane. The main function is to mediate the exchange of substances between mitochondria and cytoplasm, including small molecules and ions. Certain drugs can act on VDAC to change its permeability, leading to mitochondrial metabolic disorders and a large amount of ROS [26]. Erastin, as a ferroptosis inducer, can specifically kill tumor cells expressing RAS^V12^. It binds to and targets VDAC2/3 and can reverse the inhibition of VDAC tubule protein, then turn on VDAC. Subsequently, erastin results in *ΔΨ*m alteration, an increase in ROS in mitochondria and the occurrence of oxidative stress, eventually generating ferroptosis in cancer cells harboring RAS mutations. This may be conducive to inducing ferroptosis through the activation of the RAS–RAF–MEK pathway [27, 28]. Research has identified ferroptosis suppressor protein 1 (FSP1) independent of the classical GPX4 signaling pathway, formerly known as apoptosis-inducing factor mitochondrial 2(AIFM2), as an effective ferroptosis resistance factor, and found that the myristoylation of FSP1 is critical for inhibiting the activity of ferroptosis. FSP1 acts as an oxidoreductase, reducing coenzyme Q10 (CoQ10) and producing lipophilic free radical trapping antioxidants (RTAs), preventing the proliferation of lipid peroxides, and thereby inhibiting ferroptosis [29]. This discovery provides an important strategic reference for the evolution of drugs related to ferroptosis in cancer.

#### 2.1.3. Iron Metabolism

Iron is an indispensable component of oxygen-binding proteins (hemoglobin, myoglobin), and it coordinates the activation of mitochondrial function, DNA synthesis, and various enzymes in the regulation of immune homeostasis [30, 31]. Ferritin is a ferritin storage complex composed of ferritin light chain (FTL) and ferritin heavy chain 1 (FTH1) [32]. The accumulation of iron and lipid hydroperoxide (LPO) in cells is the basis of ferroptosis. Fe^3+^ is imported by transferrin receptor 1 (TFR1) and deposited in the endosome, where Fe^3+^ is converted to Fe^2+^. Subsequently, Fe^2+^ is released from endosomes to the cellular labile iron pool (LIP) in the cytoplasm via divalent metal transporter 1 (DMT1) [32]. The iron obtained by the body can be stored in ferritin or transported by ferritin (FPN), keeping LIP level at a low and avoiding cytotoxicity [33]. Iron can promote the formation of intracellular ROS pools through the Fenton reaction. During the Fenton reaction, iron catalyzes the decomposition of H_2_O_2_ to produce hydroxyl radicals, while promoting the oxidation of phospholipids and the degradation of membrane lipids to trigger ferroptosis [34]. Meanwhile, iron overload disrupts iron homeostasis, generating increased nontransferrin-bound iron (NTBI) and systemic inflammation through oxidative stress [35]. FPN1 is the only known cytosolic iron output protein and is mainly distributed in hepatocytes, macrophages, and intestinal cells. The degradation of FPN1 eventually leads to an increase in intracellular iron concentration followed by an upregulation of H-ferritin to enable free iron to form compound. Heptamine can counteract systemic iron overload, promote intracellular iron isolation, and reduce cellular iron outflow and intestinal iron absorption [36].

#### 2.1.4. Lipid Metabolism

Cancer cells accumulate mitochondrial cholesterol, which increases the pore-forming activity of Bax by reducing the permeability of the outer mitochondrial membrane and contributes to cell resistance to death [37–39]. Free PUFAs are substrates for the synthesis of lipid signal transducers, but they must be esterified into phospholipids and oxidized to transmit the ferroptosis signal [17]. Phosphatidyl ethanolamine (PE), with AA or its derivative epinephrine, is a key phospholipid responsible for the death of cellular iron. Acyl-CoA synthase long chain family member 4 (ACSL4) and lysophosphatidylcholine acyltransferase 3(LPCAT3) participate in the biosynthesis and remodeling of PE, activate polyunsaturated fatty acids, and affect the transmembrane characteristics of polyunsaturated fatty acids. Therefore, reducing the expression of ACSL4 and LPCAT3 can decrease the accumulation of lipid peroxides in cells, thereby inhibiting ferroptosis [23]. CRISPR-Cas9 was used to screen the cells that induced ferroptosis, and it was found that the gene synthesizing ether-phospholipid significantly regulated the ferroptosis process. The occurrence of ferroptosis could be inhibited by inhibiting the biosynthesis of ACSL4 and PUFA-ePL [40].

#### 2.1.5. Glucose Metabolism

Dipeptidyl peptidase-4 (DPP4) is a multifunctional serine protease involved in glucose control [41]. DPP4 can bind and interact with different molecules that maintain tumor growth, invasion, and metastasis. The loss of P53 can prevent the nuclear accumulation of DPP4 in CRC cells, thereby promoting the binding of the plasma membrane-related DPP4-NOX1 complex, ultimately leading to lipid peroxidation and ferroptosis [42, 43]. In addition to DPP4, SLC7A11 overexpression can promote glucose dependence in cancer cells to increase ROS levels and cell death. However, the mechanism between glucose metabolism and ferroptosis is still unclear and needs to be confirmed by further studies [44].

#### 2.1.6. Other Mechanism (P53-Mediated)

P53 is an antitumor protein that keeps DNA stable by preventing mutations. One target gene of P53^3KR^ is SLC7A11, which encodes the xCT/SCL7A11 subunit of the system X^C−^ [17]. P53 activation significantly reduces the expression of SLC7A11 at the protein and gene levels in cells. Leading to the inhibition of the X^C−^ system, inactivation of the SCL7A11 gene impaired cystine uptake and glutathione synthesis and generated lipid peroxidation and ferroptosis [45, 46]. The cell oxidative capacity was significantly reduced after activation of P53 [47].

### 2.2. Epigenetic Regulation in Ferroptosis

**Figure 2:**
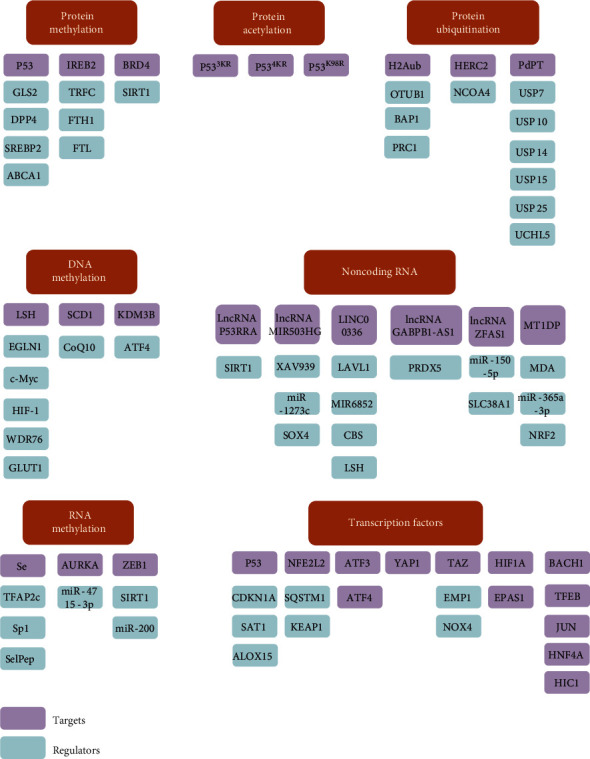
The image focused on epigenetic mechanisms of ferroptosis, which include several targets and their upstream and downstream regulatory factors (see text).

Further research on the mechanism of ferroptosis has focused on its epigenetic mechanisms (including protein methylation, acetylation and ubiquitination, and DNA methylation and RNA methylation), which regulate the role of ferritin in tumors (Figure 2). Epigenetic regulation, such as histone modification and microRNA-mediated gene silencing, plays a vital role in ferroptosis. We list the current mechanisms of epigenetic regulation of ferroptosis in tumors.

#### 2.2.1. Protein Methylation in Regulating Ferroptosis

ShRNA-mediated silencing of iron-responsive element binding protein 2 (IREB2) alters the expression of many iron genes, such as TRFC, FTH1, and FTL, thereby altering iron absorption, metabolism, and storage, which is also based on the epigenetic mechanisms and iron effects [15]. In addition to inhibiting the activity of the X^C−^ system, P53 can also mediate ferroptosis by directly targeting diamine acetyltransferase and glutaminase liver isoform 2 (GLS2), which are involved in the regulation of glutamine metabolism [43, 48]. P53 can also inhibit ferroptosis by binding to dipeptidyl peptidase 4 (DPP4) [42]. P53 may also regulate ferroptosis by mediating the mevalonate pathway. P53 promotes the expression of Lipin1 to inhibit the transcriptional activity of sterol regulatory element binding protein 2 (SREBP2), prevents the production of acetyl-CoA, and reduces the synthesis of cholesterol [37]. In addition, P53 mediates the expression of ATP-binding cassette A subfamily member 1 (ABCA1). ABCA1 is responsible for the reverse transcription of cholesterol from the plasma membrane to the endoplasmic reticulum, resulting in the inactivation of SREBP2. Ultimately, the production of squalene, coenzyme Q, and other substances promotes the process of ferroptosis [49]. Furthermore, BRD4 belongs to the bromodomain and extra terminal domain family, which can recognize acetylation sites and recruit transcription factors. The BRD4 inhibitor, JQ1, regulates proteins or histones through an epigenetic mechanism. It can inhibit the histone methylase G9a or activate the histone deacetylase SIRT1 to inhibit the expression of BRD4 to induce ferroptosis, thereby inhibiting the proliferation of tumor cells [50].

#### 2.2.2. Protein Acetylation in Regulating Ferroptosis

The GPX4 expression in cancer tissues exceeded than that in normal tissues and was negatively correlated with the prognosis of patients with all types of cancer. After the upstream regulation of GPX4 was analyzed, high GPX4 levels in cancer cells may be associated with epigenetic regulation, such as DNA methylation and histone methylation or acetylation [51]. The mutant P53^3KR^ showed an acetylation defects, which could indirectly inhibit the absorption of cysteine and reduce the consumption of GSH, which would lead to lipid peroxidation and eventually the ferroptosis [46]. Cystinase depletes cysteine, which works as a precursor to glutathione and as a precursor to metabolism in the glutathione-independent hypertrophic axis. Finally, cystinase depletion promotes ferroptosis [52]. Further research identified P53^4KR^ as the acetylation site of P53 lysine K98 in mice. Loss of K98 acetylation alone (P53 K98R) had a limited induction of P53-mediated ferroptosis, whereas P53 4KR was found in all four acetylation sites (P53 ^4KR^: K98R+3^KR^ [K117R + K161R + K162R]). P53^4KR98^ is defective in inhibiting tumor growth. The acetylation of P53 plays an important role in the process of ferroptosis and clearly inhibits the proliferation of tumor cells.

#### 2.2.3. Protein Ubiquitination in Regulating Ferroptosis

The deubiquitinase OTUB1 is often overexpressed in human cancers, and by stabilizing the cystine transporter SLC7A11, it replicates the ferroptosis process of cancer cells and promotes tumor development [53]. The tumor suppressor BAP1 is an H2A deubiquitinating enzyme that can inhibit the expression of SLC7A11 by reducing H2A ubiquitination (H2Aub) on the SLC7A11 promoter and then exert its antitumor effect by regulating ferroptosis. Both BAP1 and PRC1 (the main H2Aub ubiquitin ligase) inhibit the expression of SLC7A11 [54]. DUB inhibition can effectively treat cancer by inactivating P53 and BAP1 through inhibiting the expression of SLC7A11 or upregulating either OTUB1 or CD44 to stabilize the SLC7A11 protein, inducing proteasome inhibition and apoptosis [53]. PdPT is an inhibitor of DUBs, including USP7, USP10, USP14, USP15, USP25, and UCHL5. These inhibitors activate caspase-dependent apoptosis and GPX4-degradation-dependent ferroptosis, both of which contribute to the accumulation of ubiquitination proteins leading to cell death [55]. P53 reduces the occupancy rate of H2Bub1 in the regulatory region of the SLC7A11 gene and inhibits the expression of SLC7A11. P53 linked to ferroptosis through an epigenetic pathway mediated by H2Bub1. Overexpression of miR-17-92 or inhibition of A20 can increase the expression of ACSL4 in HUVECs, thus significantly reducing endothelin-induced cell growth inhibition and lipid peroxide production [56]. Ferritin can be degraded by two mechanisms: lysosomes and proteasomes [57]. In the case of iron deficiency, nuclear receptor coactivator 4 (NCOA4) specifically binds iron-rich ferritin to autophagosomes through FTH1 and transports it to the lysosome to release iron. At high iron concentrations, NCOA4 is ubiquitinated and degraded by the ubiquitin ligase HERC2, which affects the stability of the protein. Inhibition of NCOA4 can inhibit the degradation of ferritin and the occurrence of ferroptosis [18].

Deubiquitin provides a new mechanism of action for ferroptosis through epigenetic regulation.

#### 2.2.4. DNA Methylation in Regulating Ferroptosis

EGLN1 and c-Myc directly activate the expression of LSH by inhibiting HIF-1 inhibition. LSH, a DNA methylation modifier, interacts with WDR76 to inhibit the iron effect by activating GLUT1, a gene related to lipid metabolism, and SCD1, and FADS2, genes related to iron interaction, thereby participating in the Warburg effect [58]. Stearoyl CoA desaturase (SCD1), an enzyme that catalyze the rate-limiting step of monounsaturated fatty acid synthesis in fat, is highly expressed in ovarian cancer tissues, cell lines, and ovarian cancer stem cells. Inhibition of SCD1 induces the reduction of coenzyme Q10, which induces lipid oxidation and cell death [59]. KDM3B, a histone H3 lysine 9 demethylase, synergizes with the transcription factor ATF4 to upregulate SLC7A11 expression and prevent ferroptosis caused by erastin [60].

#### 2.2.5. RNA Methylation and in Regulating Ferroptosis

Se recruits transcription factor activation enhancer binding protein 2C (TFAP2c) and Sp1 to the GPX4 promoter region, thereby upregulating the expression of GPX4 [61]. High-level expression of GPX4 can reduce the level of cell death by inhibiting ferroptosis. Selenocysteine peptide (Tat, SelPep) can also promote cell survival by inhibiting ferroptosis [62]. Auroral kinase A (AURKA) was significantly overexpressed in upper gastrointestinal adenocarcinoma (UGC), in which the methylation level of several CpG nucleotides upstream of miR-4715-3p increased. However, 5-aza-2′-deoxycytosine can induce upstream nucleotide demethylation of miR-4715-3p in UCG cells and restore its expression, which in turn leads to AURKA downregulation, GPX4 inhibition, and cell ferroptosis [63]. Epigenetic reprogramming of epithelial-mesenchymal transformation (EMT) regulates ferroptosis in cancer cells, as cancer cells with epithelial characteristics or dense cell populations are more susceptible to ferroptosis. The decreased expression of e-cadherin or the increased expression of ZEB1 in cells leads to the transition of cancer cells to a mesenchymal state. For example, through SIRT1 induction or miR-200 inhibition, cancer cells are more sensitive to ferroptosis and prone to ferroptosis induction. In contrast, 5-azacytidine induces CDH1 demethylation, leading to the retention of epithelial characteristics of cancer cells and reduced sensitivity to ferroptosis. Therefore, EMT promotes ferroptosis in cancer cells through epigenetic regulation [64].

#### 2.2.6. Noncoding RNA in Regulating Ferroptosis

lncRNA is a class of noncoding RNA with the length of over 200 nucleotides. It does not code for proteins but binds to DNA/RNA or protein to perform its regulatory function. Gene regulation may occur as cis or trans. The functions of lncRNA include the regulation of RNA processing events (splicing, editing, localization, translation, and degradation), and its regulatory functions are related to the occurrence and development of various types of cancer. Cytoplasmic lncRNA P53RRA regulates the iron process by reducing the transcription of metabolic genes, including SCL7A11, to increase lipid, ROS, and iron concentrations. Chromatin remodeling protein, lymphoid specific helicase (LSH), and Cfp1 silencing all increased the expression of P53RRA. lncRNA P53RRA leads to a high retention rate of P53 in the nucleus, which further results in apoptosis and ferritin action [65]. In non-small-cell lung cancer (NSCLC), the downregulation of lncRNA MIR503HG induced by XAV939 may inhibit tumor growth by sponging miR-1273c and regulating SOX4 expression [66]. LINC00336, a nuclear lncRNA, has been upregulated as a competitive endogenous RNA in lung cancer. It binds to the RNA nucleotides 1901-2107 through the nucleotide binding protein ELAVL1 (similar to ELAV RNA binding protein 1), thereby inhibiting ferroptosis. Meanwhile, LINC00336 acts as an endogenous sponge for microRNA 6852 (MIR6852), which regulates the expression of cysteine synthase (CBS) (a surrogate marker for fertilized egg formation), while MIR6852 inhibits cell growth by promoting ferroptosis. In addition, LSH can increase the expression of ELAVL1 through the P53 signaling pathway, and ELAVL1 improves the expression of LINC00336 by stabilizing the posttranscriptional level. lncRNA and ceRNA networks can regulate tumorigenesis and the induction of ferroptosis [67]. Globin upregulates lncRNA GABPB1-AS1, which downregulates GABPB1 levels by blocking GABPB1 translation, thus leading to the downregulation of the peroxiredoxin-5 (PRDX5) peroxidase gene and ultimately inhibiting the antioxidant capacity of cells. lncRNA can regulate the process of oxidative stress in cells and cause ferroptosis [68]. The inhibitory effect of lncRNA ZFAS1 eliminated lipid peroxidation induced by BLM (bleomycin). By acting as a ceRNA and sponge for miR-150-5p to downregulate SLC38A1 expression, silencing lncRNA ZFAS1 can reduce ferroptosis [69]. Metallothionein 1D pseudogene (MT1DP) is a kind of lncRNA that can aggravate oxidative stress by inhibiting antioxidant effects. Ectopic MT1DP upregulates the levels of malondialdehyde (MDA) and ROS. In cancer cells exposed to protein, the intracellular ferrous ion concentration increases, and the GSH level decreases. MT1DP regulates the expression of NRF2 by stabilizing miR-365a-3p, and downregulating NRF2 makes cells sensitive to ferroptosis caused by protein kinases [70]. In addition, miR-214 can bind to the 3′ untranslated region (3′UTR) of PVT1, P53, or TFR1. lncRNA PVT1 regulates ferroptosis through miR-214-mediated TFR1 and P53 [71].

#### 2.2.7. Transcription Factors in Regulating Ferroptosis

Several transcription factors (such as P53, NFE2 L2, ATF3, ATF4, YAP1, and TAZ) play multiple roles in the regulation of ferroptosis sensitivity. Cyclin-dependent kinase inhibitor 1A (CDKN1A) can affect cell cycle to induce apoptosis and iron death [72]. Spermidine/spermine N1-acetyltransferase 1 (SAT1) can increase the expression of ALOX15 and induce arachidonic acid peroxidation [48]. P53 regulates the ferroptosis by activating the P53-CDKN1A and p53-SAT1-ALOX15 pathways. NFE2L2 generally functions as a master negative regulator of ferroptosis, because activation of the SQSTM1–KEAP1–NFE2L2 pathway is an important homeostasis mechanism to block ferroptosis in hepatocellular carcinoma (HCC) induced by sorafenib. Activating transcription factor 3 (ATF3) and activating transcription factor 4 (ATF4) are markers of endoplasmic reticulum stress that are upregulated to promote ferroptosis. Phosphorylated yes-associated protein 1 (YAP1) can induce ferroptosis, and transcriptional coactivator with PDZ-binding motif (TAZ) regulates the sensitivity of ferroptosis via the TAZ–EMP1–NOX4 pathway. Transcription factor AP-2 gamma (TFAP2C) and specificity protein 1 (SP1) increase the GPX4 expression to limit ferroptosis. Hypoxia-inducible factor 1 alpha (HIF1A) inhibits apoptosis and resists ferroptosis. Endothelial PAS domain protein 1 (EPAS1) enriches lipids to induce ferroptosis. Other transcription factors that regulating ferroptosis include BTB domain and CNC homolog 1 (BACH1), transcription factor EB (TFEB), Jun proto-oncogene, AP-1 transcription factor subunit (JUN), hepatocyte nuclear factor 4 alpha (HNF4A), and HIC transcriptional repressor 1 (HIC1) which all have unclear molecular mechanism [41].

## 3. Tumor Therapy Based on Nonepigenetic and Epigenetic of Ferroptosis

Specific induction of ferroptosis in tumor cells has become a novel target for tumor therapy. Ferroptosis induced by small molecular compounds can evade chemotherapeutic drug resistance and induce the death of cancer cells under the condition that some classical chemotherapeutic drugs have no effects [4]. The application of nanobiology technology in the efficient delivery of antitumor drugs conforms to the biological characteristics of tumors to a certain extent with great advantages in the efficiency of drug delivery. In recent years, research has increasingly focused on cross-disciplinary approaches, where nanodelivery systems combine the advantages of small molecules that induce ferroptosis (drug targeting and attenuating drug resistance in cancer cells) to treat tumors at the epigenetic or metabolic level [73]. In the following sections, we review emerging nonepigenetic-based therapies (nanotherapeutics) and the epigenetic-based mechanisms of tumor therapy potential.

### 3.1. Nonepigenetic Mechanism (Focus on Optical Nanotherapeutics)

#### 3.1.1. Amino Acid Metabolism-Mediated

Small molecules targeting amino acid metabolism include ferroptosis inhibitors of system X^C-^, competitive inhibitors of SLC7A11, and immune-targeting SLC7A11. Inhibitors of system X^C−^, include erastin, salazosulfapyridine, and sorafenib, can induce ferroptosis in certain tumor cells. Capsazepine (CPZ), a competitive inhibitor of SLC7A11, can inhibit the exchange of L-cystine and L-glutamate in triple-negative breast cancer cells through system X^C−^ to promote ferroptosis. For immune-targeting SLC7A11, several vaccines (such as DNA-based, VLP-based, and BoHV-4-based vaccines) against breast cancer have been tested in vivo [74].

#### 3.1.2. Mitochondrial Mechanism-Mediated

In hundreds of cancer cell lines, the expression of FSP1 is positively correlated with the drug resistance of ferroptosis. And FSP1 mediates resistance to ferroptosis in lung cancer cells and mouse tumor xenografts [29].

#### 3.1.3. Iron Metabolism-Mediated

Bortezomib can inhibit the upregulation of iron by ferritin and reduce ROS, thus inhibiting ferroptosis. Reducing basic ferritin levels or supplementing iron can reverse bortezomib resistance. In addition, by targeting the downregulation of ferritin by miR-133a, drug-resistant breast cancer cells can partially restore sensitivity to cisplatin and Adriamycin [75]. Regulating iron metabolism contributes to overcoming cancer resistance and improving curative effects. Artesunate (a drug used to treat malaria) has additional antitumor and antiviral activities [76]. It induces iron dependence and oxidative stress reduction in PDAC cell lines and is blocked by the iron chelating agents deferramine or ferrostatin-1 [77].

#### 3.1.4. Lipid Metabolism-Mediated

Lipid metabolism-mediated ferroptosis depends on the biosynthesis of PUFA-containing phospholipids, ACSL4, and LPCAT3. Small molecules of FIN56 [18], FINO2 [78], buthionine-(S,R)-sulfoxime (BSO) [79], RSL3, DPI19, DPI18, DPI17, DPI13, DPI12, DPI10 (ML210), DPI7 (ML162), and the anticancer agent hexamethylmelamine [15, 21, 80, 81] are inhibitors of GPX4 to lead the accumulation of PUFA hydroperoxides and tumor cell ferroptosis. Statins were found to cause GPX4 to be downregulated as a result of isoprenol pyrophosphate depletion; in fact, its mechanisms are similar to chemical GPX4 inhibitors [12]. Artesunate can induce GSH depletion and lipid peroxidation and selectively induce ferroptosis of head and neck cancer (HNC) cells without damaging normal cells [82]. The ferroptosis and the increase in lipid ROS by dihydroartemisinin (DHA) can be reversed by the ferroptosis inhibitor ferrostatin-1, and the main target is GPX4 [83].

#### 3.1.5. Optical Nanotherapeutics

**Figure 3:**
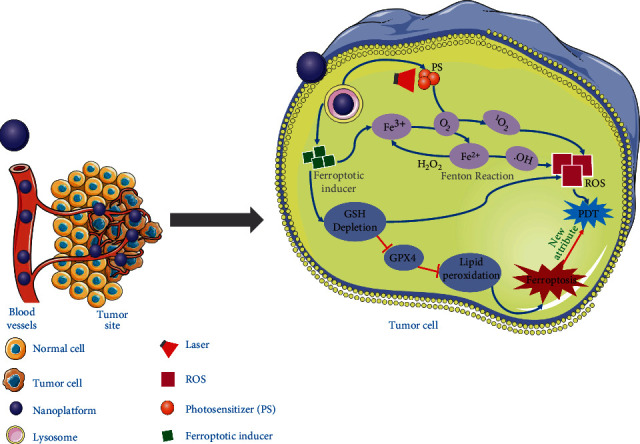
Schematic illustration of optical nanotherapeutics based on ferroptosis for tumor therapy (dependent on PDT and nanoplatforms).

Optical therapy based on nanomaterials, including photothermal therapy (PTT), photodynamic therapy (PDT), and photochemotherapy [84]. Nanoplatforms mainly include metal nanomaterials, carbon-based nanomaterials, especially organic nanomaterials (for biosafety). Several nanomaterials loaded with chemotherapy drugs have been used in clinical therapy, such as docetabine (adriamycin liposome) [85, 86]. Near-infrared (NIR) light combined with nanomaterials can penetrate tissues and convert invisible light into visible light of corresponding wavelengths. Meanwhile, the mild hyperthermia induced by NIR can also increase the vascular permeability of tumor tissues with novel blood vessels, thereby bringing specific drug accumulation and enhanced cytotoxicity [87]. However, the disadvantage of NIR is also the thermal effect of the tissue, which causes thermal damage to the tissue. By controlling the external illumination energy and location, NIR can finely locate the nanoplatform combined with the ferroptosis inducer in the tumor area. The iron ion concentration of a considerable number of cancer cells is significantly higher than that of the corresponding nontumor cells. For example, the serum iron level of human breast cancer patients is significantly higher [88]. Therefore, inducing ferroptosis of these cells may be a potentially effective targeted therapy. The discovery of potent ferroptosis inducers may explore a novel way to treat a variety of treatment-resistant tumors. Nanoplatforms loaded with powerful ferroptosis inducers combined with optical technology inducers can be directed to the tumor site by increasing the retention (EPR) effect through passive targeting or active targeting of surface-binding molecules [89], which supports eliminating drug-resistant cancer cells **(**Figure 3**)**.

Doxorubicin (Dox) contained in mesoporous carbon nanoparticles can induce ferroptosis of various tumor cells, including breast cancer cells and human cervical cancer (HeLa) cells [90]. Iron-based nanoparticles can induce ferroptosis and inhibit tumor cell proliferation. The mechanism is to release Fe^2+^ and Fe^3+^ in acidic lysosomes to increase the concentration of iron ions [91]. Ferroptosis inducers can also be used as research tools. In 2019, Hirayama successfully established an imaging method that can monitor the specific Fe^2+^ of each organelle and explore the iron ion concentration in mitochondrial lysosomes and endoplasmic reticulum organelles during the process of ferroptosis mediated by the erastin pathway [92]. A novel probe (a fluorescent probe based on quinoxalinone) for the observation of the ferroptosis phenotype can be used to identify ferroptosis [93].

PDT is a noninvasive or minimally invasive therapy for tumor therapy through the combined action of light, photosensitizer, and oxygen molecules in tissues, which is a novel medical discipline. PDT combined with an oxygenation strategy has been widely used in tumor therapy. Targeted photosensitizers are used in lesions to accept the corresponding wavelength of visible optical irradiation. After stimulation, the energy transfer, and electron transfer process, the surrounding oxygen molecules with chemical properties of singlet oxygen, superoxide free radicals, and reactive oxygen species, such as material and substrate molecule free radicals, are applied to biological macromolecules, eventually lead to death or apoptosis of tumor cells [94]. The fully active MOF triggers the ferroptosis mechanism to facilitate photodynamic antitumor therapy (a fully active MOF nanocarrier contains disulfide, which is used to encapsulate the photosensitizer Ce6). Regardless of light exposure, Ce6-loaded nano-corporator consumed intracellular GSH in mouse breast cancer cells (4T1) through a disthio-mercapto exchange reaction. High reactive singlet oxygen in PDT can deplete GSH, and GSH depletion leads to the inactivation of GPX4 and enhances the cytotoxicity of ferroptosis to a degree controlled by the redox reactive nanocarrier [95]. A novel multimode therapeutic agent is FePt/BP-PEE-FA nanoplatform, in which FePt nanoparticles (FePt NPs) are loaded onto ultrathin black phosphorous nanotablets (BPNs). Under laser irradiation at different wavelength, BPN produces photothermal and photodynamic changes. As a proliferative agent, FePt NPs can transform endogenous H_2_O_2_ into ROS through the Fenton reaction, which eventually leads to cell death. The nanoplatform can inhibit tumor growth through synergistic treatment and can control the growth of primary and untreated distant tumors, making it a multifunctional composite nanopreparation with effective antitumor application potential [96].

Based on MnO_2_@HMCu2-xS nanocomposite material (HMCM), a photothermal (PT) and autophagy enhanced ferroptosis treatment method was constructed to achieve effective tumor ablation. HMCM has PT-enhanced GSH depletion ability, which induces PT-enhanced ferroptosis through enhanced inactivation of GPX4. Meanwhile, the release of Mn^2+^ in response to GSH can generate ROS through a Fenton-like reaction to enhance intracellular oxidative stress caused by the accumulation of LPO in ferroptosis [97]. Mesoporous carbon nanoparticles (MCNs) are oxidization-resistant drug carriers that carry Dox as chemotherapeutic drugs and combine with heat-sensitive CO precursors to form novel nanoplatforms. FeCO can absorb near-infrared light and convert it into enough heat to trigger the release of CO. The CO molecules generated can increase the sensitivity of cancer cells to chemotherapy drugs through the ferroptosis pathway and release Dox in the acidic tumor microenvironment [90]. Photoacoustic (PA) imaging guided second near-infrared photothermal iron therapy with high photothermal conversion efficiency hybrid semiconductor nanoenzyme (HSN). Second near-infrared light can also reduce the thermal effect of irradiating tissue. HSN contains an amphiphilic semiconducting polymer as a light-to-heat converter, a PA emitter, and an iron chelated Fenton catalyst. Under light irradiation, HSN not only generates heat to induce cytotoxicity but also enhances the Fenton reaction. The increase in ·OH production not only promotes ferroptosis but also promotes cell apoptosis [98]. PA imaging guides photothermal ferroptosis therapy through iron chelating semiconductor polymer nanoparticles (SPFeNs). In the acidic tumor cell microenvironment, SPFeNs can generate hydroxyl free radicals, leading to ferroptosis. Combined with near-infrared laser irradiation, local tissues generate much heat, which accelerates the ferroptosis process. This combination of photothermal therapy and iron can reduce the dose of iron and effectively inhibit tumor proliferation [99].

Nanodrug release system (Fe^3+^ crosslinked oxidized starch + upconversion nanoparticle UCNPs), UCNPs can resist the shortcomings of the limited penetration depth of near-infrared light and can reduce Fe^3+^ to Fe^2+^. As the valence of the compound changes the gel network, it is destroyed and eventually leads to the release of Fe^2+^ and Dox. The Fenton reaction between Fe^2+^ and H_2_O_2_ produces a mass of ROS to promote cell ferroptosis, and the same released Dox as a chemotherapeutic drug can penetrate the nucleus to induce cell apoptosis. Traditional chemotherapeutic drugs are equipped with the ferroptotic effects, and combined therapy can significantly improve the antitumor efficacy [100]. Targeted hormone therapy has little effect on triple-negative breast cancer (TNBC), but azo-CA4 can lead to microtubule breakdown and cell cycle arrest at the G_2_/M stage, which provides the possibility for TNBC anticancer treatment. The nanocarrier loaded with azo-CA4 reduced Fe ^3+^ to Fe ^2+^ after being irradiation with near-infrared light, promoted lipid peroxidation to induce ferroptosis, and induced TNBC cell death through apoptosis and ferroptosis [101]. In the nano-photosensitizer complex (CSO-BHQ-IR780-Hex/MIONPs/Sor), CSO induced the whole complex to lyse, and IR780-HEX, MIONP, and sorafenib were dispersed in the cells. IR780-hex can anchor on the mitochondrial membrane. After near-infrared irradiation, the intracellular iron concentration increases, and the mass of lipid peroxides increases, accelerating the process of cell ferroptosis. The localization effect of the nanometer platform showed an excellent tumor targeting effect and showed excellent tumor suppressive effect in the simulated mouse model of breast tumor [102]. Epithelial-mesenchymal transformation (EMT) of cells is a famous characteristic of drug-resistant cancer, but these cells are sensitive to the ferroptosis process. Nanorods cause superparamagnetic iron oxide nanoparticles (SPIONs) to target and aggregate into tumor cells, releasing iron ions in an acidic lysosomal environment [103]. Hemoglobin (Hb) was connected with photosensitive chlorin e6 (Ce6), and a nanoplatform (SRF@Hb-Ce6) was constructed by using sorafenib (SRF), which combined with oxygen to enhance PDT and ferritin. Iron binds to oxygen, and hemoglobin provides both oxygen for oxygen-dependent PDT and iron for iron-dependent death. SRF@Hb-Ce6 enhances PDT and induces ferroptosis. In addition, PDT enhances ferritin regulation by recruiting immune cells. Synergistic therapy shows the advantages of PDT and ferrite combined with nanoplatforms, which may become a novel direction of tumor therapy [104]. SLC7A11 is upregulated in oral squamous cell carcinoma (OTSCC), excessive intracellular ROS accumulation, increased O_2_ concentration, and inhibition of SLC7A11 expression, leading to postirradiation induced ferroptosis, a nanomedicine formed by the combination of the photosensitive agent Ce6 and thickening inducer erastin, and produces good antitumor effects in transplanted tumor mouse models with low other tissue cytotoxicity [105].

Because ultrasound is convenient and noninvasive and has a high tissue penetration performance, the emerging acoustic dynamic therapy (SDT) can break through the limited tissue penetration efficiency of light and, at the same time, as a noninvasive therapy, solve the existing problems in tumor therapy [106, 107]. SDT can solve the problem of low tissue penetration depth in phototherapy, but its application has many limitations. However, it can be used to regulate the tumor microenvironment with catalytic nanodrugs to enhance the effect of SDT. MnO_X_ combined with a nanoultrasound sensitizer is used to convert tumor overexpressing H_2_O_2_ molecules into oxygen and promote the production of SDT-induced reactive oxygen species. Moreover, the tumor has good histocompatibility, high biosafety, and easy metabolism, which provides a feasible new way to study nanoscale tumor dynamics [108].

### 3.2. The Possibility and Prospect of Epigenetic Regulation in Tumor Therapy

At present, the mainstream-targeted anticancer drugs that have been applied in the clinical environment induce apoptosis of cancer cells through signal transduction pathways, while small molecules, nanomaterials, and epigenetic regulation based on ferroptosis in recent studies have shown great potential for antitumor application. Epigenetics plays a vital role in the biological process of cancer. Recent studies have focused on the epigenetic regulation of ferroptosis, including a variety of modifications. Of which ncRNA is mainly based on the regulation of GSH and iron metabolism in ferroptosis, but the specific regulatory mechanism has not yet been explored [109]. Research on ferroptosis based on epigenetic mechanisms is still in the stage of screening and verifying the regulatory effect, and there is still a long way to go for clinical application. However, with the improvement of the epigenetic regulatory network, many kinds of regulatory factors can be used as targets for ferroptosis of cancer cells and become novel treatment strategies for drug-resistant cancer. Because ferroptosis also exists in normal cells, combined with emerging technologies such as nanometers, it may become a novel direction for targeted drug therapy. The advantages of various fields can be combined to enhance the therapeutic effect and reduce the occurrence of adverse events.

## 4. Summary and Conclusions

The epigenetic regulation of ferroptosis is currently not fully understood, which limits its application to cancer treatment. First, to ensure that epigenetic modifications cause cancer cells to die because of iron instead of other death methods, we should continue to study various modification methods. Second, expand the research regulatory network and screen out specific markers that target certain cancers with different cancer profiles. Third, the regulation of epigenetics is not limited to researching phenomena and screening ncRNAs. It should be repeated in vivo and in vitro experimental verification, combined with emerging technologies to promote clinical application research; finally, reducing the adverse reactions of other organs and tissues should be considered. Regulation should be specific to cancer cells.

In addition, the combination of multiple therapeutic drugs or methods has shown obvious advantages in clinical cancer treatment. Chemotherapy and targeted agents are still the most common methods to inhibit tumor growth, and the main form of cell death induced by chemotherapy is apoptosis. Due to the emergence of acquired drug resistance, certain cancer cells escape apoptosis and are resistant to the above therapies. A variety of invasive cancers, which are resistant to chemotherapy and targeted agents, are susceptible to nonapoptotic cell death form ferroptosis [41]. Despite the rapid development of cancer therapies based on ferroptosis, the road to clinical application is still far away, and the potential side effects and limitations pose great challenges. First, the specificity of tumor therapy remains. Second, the potential toxicity and side effects of rust inducers should be fully studied to ensure the tumor-specific trigger of Fenton reaction and avoid off-target toxicity to normal tissues. Finally, in addition to the convenience brought by nanotherapy, the biosafety issues, complexity of materials, adaptability of different patients, and forms of application need to be further explored to achieve the optimal treatment effect and reduce adverse reactions. For optical therapy, it is possible to seek for II-zone near-infrared light with longer wavelength, which can reduce tissue damage produced by NIR while retaining tissue penetration. NIR is less toxic to normal cells and tissues with low light exposure doses. Meanwhile, we explored the lowest light energy when the drug accumulates at the maximum concentration and found a relatively safe illumination mode. The combination of optics and material science promotes clinical conversion applications, and the combined treatment system provides the next generation of iron-promoting therapy for antimalignant tumors. The use of ferroptosis inducers to overcome the resistance of tumor cells to chemotherapy has become a hot spot. In addition, nanoplatforms combined with optical technology to target release inducers to reduce systemic toxicity provide a more efficient and safe treatment for tumor. The implementation of ferroptosis driven optical nanotherapeutics in cancer treatment mainly depends on PDT and nanoparticle platforms. Although no relevant clinical trial has been reported, optical nanotherapeutics based on the mechanism of ferroptosis are potential enough. To the best of our knowledge, PDT has been applied in treating malignant skin tumors (such as Bowen's disease, Paget's disease, basal cell carcinoma, and squamous cell carcinoma) and precancerous skin diseases in clinical practice [110]. Moreover, therapeutic nanoparticle (NP) platforms, including liposomes, albumin NPs, and polymeric micelles, are under clinical research [111]. Hence, we consider that optical nanotherapeutics based on the mechanism of ferroptosis are a promising novel treatment strategy in tumor treatment. Similar to other scientific advances that have revolutionized medicine in the past few decades, optical nanotherapeutics must be mature in order to be fully functional.
